# New Estimation of Antibiotic Resistance Genes in Sediment Along the Haihe River and Bohai Bay in China: A Comparison Between Single and Successive DNA Extraction Methods

**DOI:** 10.3389/fmicb.2021.705724

**Published:** 2021-09-20

**Authors:** Chao Wu, Guicheng Zhang, Wenzhe Xu, Shan Jian, Liyin Peng, Dai Jia, Jun Sun

**Affiliations:** ^1^Tianjin Key Laboratory of Marine Resources and Chemistry, Tianjin University of Science and Technology, Tianjin, China; ^2^Research Centre for Indian Ocean Ecosystem, Tianjin University of Science and Technology, Tianjin, China; ^3^College of Marine Science and Technology, China University of Geosciences, Wuhan, China

**Keywords:** antibiotic resistance genes, sediment, DNA extraction, DNA yield, pathogenic bacterium

## Abstract

Sediment is thought to be a vital reservoir for antibiotic resistance genes (ARGs). Often, studies describing and comparing ARGs and their potential hosts in sediment are based on single DNA extractions. To date, however, no study has been conducted to assess the influence of DNA extraction efficiency on ARGs in sediment. To determine whether the abundance of ARGs is underestimated, we performed five successive extraction cycles with a widely used commercial kit in 10 sediment samples collected from the Haihe River and Bohai Bay. Our results showed that accumulated DNA yields after five extractions were 1.8–3.1 times higher than that by single DNA extractions. High-throughput sequencing showed that insufficient DNA extraction could generate PCR bias and skew community structure characterization in sediment. The relative abundances of some pathogenic bacteria, such as Enterobacteriales, Lactobacillales, and Streptomycetales, were significantly different between single and successive DNA extraction samples. In addition, real-time fluorescent quantitative PCR (qPCR) showed that ARGs, *intI*1, and 16S rRNA gene abundance strongly increased with increasing extraction cycles. Among the measured ARGs, sulfonamide resistance genes and multidrug resistance genes were dominant subtypes in the study region. Nevertheless, different subtypes of ARGs did not respond equally to the additional extraction cycles; some continued to have linear growth trends, and some tended to level off. Additionally, more correlations between ARGs and bacterial communities were observed in the successive DNA extraction samples than in the single DNA extraction samples. It is suggested that 3–4 additional extraction cycles are required in future studies when extracting DNA from sediment samples. Taken together, our results highlight that performing successive DNA extractions on sediment samples optimizes the extractable DNA yield and can lead to a better picture of the abundance of ARGs and their potential hosts in sediments.

## Introduction

Antibiotics are one of the most important medical advances and are widely used in clinical medicine, plants, livestock, and aquaculture (Hu et al., 2003; Cromwell, 2006). In recent years, however, the abuse and excessive discharge of antibiotics have led to a mass of antibiotic-resistant bacteria emerging in the natural environment (Davies, 1994). More seriously, the drug resistance of pathogenic bacteria is increasing, producing plentiful multiple drug-resistant strains and even superbugs such as “NDM-1” with resistance to the vast majority of antibiotics (Walsh et al., 2011). The emergence of superbugs is mainly caused by recombination or mutation of antibiotic resistance genes (ARGs), which have the ability to inactivate antibiotics (Davies and Davies, 2010). Currently, ARGs have been identified as emerging contaminants due to their characteristics of environmental persistence and eco-environmental risk (Pruden et al., 2006).

Recent studies have verified that ARGs are ubiquitous in most natural environmental media, including sewage (Zhu et al., 2013), atmosphere (Liang et al., 2020), soil (Riesenfeld et al., 2004), biofilms (Guo et al., 2018), sediment (Zhu et al., 2017; Guo et al., 2020; Zhao et al., 2020), and drinking water (Han et al., 2020), among which natural aquatic environments, including water phases and sediments, are thought to be an ideal reservoir for the evolution, spread, and gene transfer of ARGs (Luo et al., 2010; Zhao et al., 2020). On a large scale, ARGs and antibiotic-resistant bacteria (ARB) are most abundant and diverse in urban rivers, followed by lakes, reservoirs, estuaries, and coastal environments (Li et al., 2018). This is because urban rivers receive various pollutants from domestic settlements, hospitals, industries, livestock, and aquaculture, and these pollutants are gradually diluted from rivers to estuaries and finally to coastal environments (Lu et al., 2021). Compared with the water phase, sediment is thought to be a key hotspot for the enrichment and dissemination of ARGs (Luo et al., 2010; Guo et al., 2020; Zhang et al., 2020). It has been demonstrated that the diversity and abundance of ARGs in sediment in some regions even exceed those in the water phases (Luo et al., 2010; Zhang et al., 2018). Considering the important function of enriching and keeping ARGs in sediment, it is, therefore, essential to build a series of unified standards to guarantee the accuracy of quantifying ARGs in sediment.

Traditionally, the study of ARGs and their potential hosts in sediment is based on single DNA extraction by the kit method or lab method (Luo et al., 2010; Mao et al., 2013; Zhu et al., 2017; Zhang et al., 2020). However, recent evidence has shown that single DNA extraction may greatly underestimate the DNA yield in soils and sediments, and bacterial diversity may be biased due to insufficient DNA extraction (Feinstein et al., 2009; Han et al., 2013; Dimitrov et al., 2017). A more recent study indicated that insufficient DNA extraction of swine manure may underestimate the abundance of ARGs and their potential hosts (Luo et al., 2021). Compared with swine manure, the composition of sediment is more complex, containing not only different sizes of fragments ranging from silt to boulder but also many dead or living organisms. To date, however, no study has been conducted to assess the influence of DNA extraction efficiency on ARGs and their potential hosts in sediment. If this speculation is verified, ARGs and their hosts may be greatly underestimated and should be reappraised in sediment.

The Haihe River, the largest river system in northern China, flows through an urban city and an agricultural area before discharging into Bohai Bay (Luo et al., 2010). Numerous studies related to ARGs have been conducted in the main streams, tributaries, and estuary area of the Haihe River and showed that the ARGs in the sediment presented high eco-environmental risk, especially the sulfonamides and multidrug resistance genes (Luo et al., 2010; Mao et al., 2013; Dang et al., 2017; Yang et al., 2017; Zhang et al., 2020). Compared with the Haihe River, Bohai Bay is less studied but occupies an important geographical location because it is a reservoir of various pollutants from terrestrial sources. The few studies conducted in the coastal areas of Bohai Bay showed that sulfonamides and tetracycline resistance genes were highly abundant in the bay (Zhang et al., 2018, 2020). As above, although several studies have been conducted in the study region, no study has been conducted to assess the influence of DNA extraction efficiency on ARGs in sediment. To determine whether ARG abundance is underestimated, we collected sediment samples along the Haihe River and the Bohai Sea to investigate the ARGs and bacterial communities. Single and successive extraction methods based on a commercial kit were all used in the present study to extract DNA. High-throughput sequencing and real-time fluorescent quantitative polymerase chain reaction (qPCR) assays were also used in our study to investigate the composition of bacterial communities and the abundance of the main ARG subtypes, respectively.

## Materials and Methods

### Study Location and Sample Collection

**FIGURE 1:**
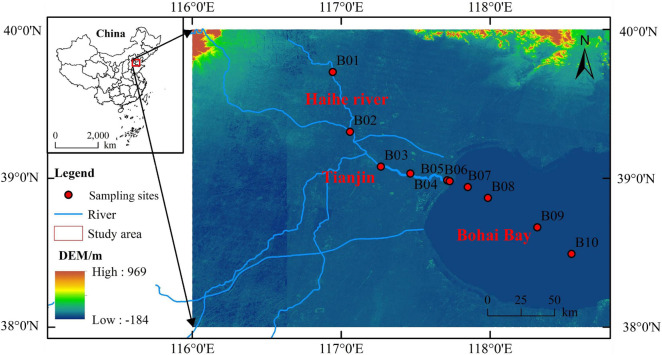
Map showing sampling stations in the Haihe River and Bohai Bay. The miniature map at the top left describes the sampling region in China. In the enlarged map, sediment samples were collected at 10 stations (red dots), among which five stations were located in the Haihe River (B1∼B5), and five stations were located in Bohai Bay (B6∼B10).

Sediment samples were collected from 10 stations along the Haihe River and the Bohai Bay in July 2020, including five sediment samples along the Haihe River and five sediment samples from the coastal to the open region of the Bohai Bay (Figure 1). The sediment samples were all collected by grab buckets following a previous method (Zhao et al., 2020). The collected samples were all kept in Ziploc bags and stored at −20°C until analyzed. In the lab, subsamples were taken from each Ziploc bag and lyophilized by a lyophilizer to calculate the moisture content in the sediments. According to the moisture content of the sediment, duplicate subsamples, which are equivalent to 0.2 g per unit dry weight was taken from each Ziploc bag for further DNA extraction.

### DNA Extraction and Quality Control

**FIGURE 2:**
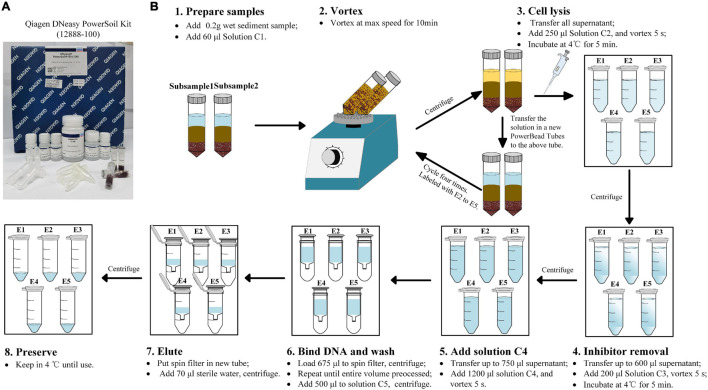
The modified procedure of DNA extraction method. **(A)** The Qiagen DNeasy Powersoil kit (12,888–100) used in the present study. **(B)** The modified procedure of the DNA extraction method used in the present study. E1∼E5 mean extraction cycles.

The genomic DNA of sediment samples were extracted by the Qiagen DNeasy Powersoil^®^ kit (12,888–100). A detailed protocol is shown in Figure 2. Briefly, the main protocol to extract DNA followed the instructions of the manufacturer, including preparation of the sample, vortex, cell lysis, inhibitor removal, bind DNA, wash, elute, and preserve. For each sediment sample, duplicate subsamples were prepared in two PowerBead tubes and vortexed concurrently. After centrifugation, the supernatant in duplicate tubes were mixed in one collection tube for further cell lysis. This procedure was labeled with single DNA extraction (E1). The above PowerBead tubes with sediment samples were not discarded and then repeatedly used to extract DNA successively. A new solution was taken from new PowerBead tubes and was replenished on the above tubes. The PowerBead tubes with sediment were extracted again for an additional four times and labeled with E2–E5. The followed operating steps were the same as those of the instructions of the manufacturer. The quantity and quality of the extracted DNA were checked using an ND-2000 NanoDrop spectrometer (Thermo Fisher Scientific, Wilmington, DE, United States). DNA samples were preserved at −80°C until analyzed.

### High-Throughput Sequencing, Quality Control, and Sequencing Data Processing

The V3–V4 variable regions of the 16S rRNA genes were amplified in the present study to reveal the total microbial composition. All the above E1 DNA samples were used as DNA templates. In addition, combined DNA samples (5 μl of DNA sample was taken from the E1–E5 tubes to combine a new DNA sample) were used in our study to show the microbial difference between single DNA extraction (E1 samples) and succession DNA extraction (total samples). The polymerase chain reactions (PCRs) were amplified with the pairwise common primer 343F and 798R using a Bio-Rad thermocycler (Bio-Rad, Redmond, WA, United States). The reagent formula and PCR procedure are given in a previous study (Lv et al., 2017). After amplification, all PCR products were verified by 1.8% agarose gel electrophoresis, and products with approximately 465-bp bands were thought as effective amplification. The following PCR product purification, quantification, and sequencing were shown in our previous study (Wu et al., 2019). All libraries were constructed and sequenced at Shanghai OE Biotech Co., Ltd. (Shanghai, China) via paired-end chemistry on an Illumina Miseq platform (Illumina, San Diego, CA, United States). The raw sequencing data obtained from the present study have been submitted to the NCBI Sequence Read Archive (SRA) with accession no. PRJNA724916.

The bioinformatics analysis was achieved using the open-source software pipeline QIMME 2 (Bolyen et al., 2019). A detailed procedure of the bioinformatics analysis can be seen in our previous studies (Wu et al., 2019). Briefly, the downstream analysis includes quality controlling (Bolger et al., 2014), assembling (Magoč and Salzberg, 2010), and denoising (Edgar et al., 2011). After denoising, the remaining effective tags were clustered at a 97% similarity cutoff to generate operational taxonomic units (OTUs) by the open-source pipeline VSEARCH (Torbjørn et al., 2016). In addition, representative sequences were also selected from the clustered OTUs in the QIMME 2 pipeline (Christian et al., 2013). The representative sequences were aligned, annotated, and blasted against SILVA v123 through the RDP classifier (Quast et al., 2013). Subsequently, a random resampling was conducted based on the OTU table to homogenize sequences across samples. The following statistical analysis are all based on the resampled OTU table.

### Real-Time Fluorescent Quantitative PCR Assay

According to previous studies, 11 ARGs were chosen and quantified in the present study for their high abundance in the study region (Luo et al., 2010; Zhao et al., 2020). The 11 ARGs were quantified by SYBR green dye method using an ABI Step One Plus Real-Time PCR System (Applied Biosystems, Foster City, CA, United States) in our study, including one 16s rRNA gene, one universal class I integron-integrase gene (*intl*I), and nine ARGs. The primers and corresponding annealing temperatures of each qPCR reaction are listed in Supplementary Table 1. Briefly, the nine ARGs in this study belong to six types of ARGs, including sulfonamide-resistant genes (*sul*1, *sul*2), tetracycline-resistant genes (*tet*M, *tet*W), beta_lactamase-resistant genes (bla_TEM_), multidrug-resistant genes (*fl*oR, qacEΔ1-01), and macrolide lincosamide–streptogramin B (*ere*A, *erm*B). In addition, the 16S rRNA gene was also measured and used as an internal control for data normalization.

For all qPCRs, triplicate 10-μl reactions were performed with 5 μl of TB Green Premix ExTaq II (Tli RNaseH Plus, Takara, Tokyo, Japan), 0.2 μM of the forward and reverse primers, 0.2 μl of 50 × ROX reference dye, 1 μl of template DNA, and 3.8 μl of nuclease-free water. The thermal cycling conditions for qPCR reactions were: predenaturation for 30 s at 95°C, followed by 45 cycles of denaturation (45 s at 95°C), annealing (30 s at different annealing temperatures, Supplementary Table 1), a melting curve analysis at 95°C for 15 s, and, finally, annealing at 60°C for 1 min. Standard curves were determined by analyzing 10-fold serial dilutions of the target gene inserted to plasmids with the final gene copy numbers ranging from 10^2^ to 10^8^ for each reaction. The *R*^2^-values of each standard curve were greater than 0.98, and PCR amplification efficiency ranged from 90 to 110% (Wu et al., 2019). The gene copies of each ARGs types were calculated based on the mean Ct values and the corresponding standard curves. In addition, non-target templates were also tested at the same conditions in the present study, and the gene copies less than 10 or undetectable were considered contamination free.

### Statistical Analysis

Alpha-diversity indices, including the Chao1 richness estimator, Ace richness estimator, Shannon diversity indices, and Simpson diversity index, were calculated based on the resampled (operational taxonomic unit) OTU table using the “vegan” package in R v3.6.2 software (R Foundation for Statistical Computing, Vienna, Austria). The UpSet plot was applied in the present study to show the intersection of OTUs between single DNA extraction and total DNA extraction using the “UpSetR” package in R v3.6.2 software (Conway et al., 2017). Rarefaction curves were also calculated in our study using PAST3 software and visualized with Origin v8.5 software.

Beta diversity was measured by using the Bray–Curtis dissimilarity. The Bray–Curtis dissimilarity between each pair of samples was first calculated using the “Vegan” package of the R v3.6.1 software. Subsequently, the distance decay of the bacterial communities was fitted between the geographic distance and the Bray–Curtis dissimilarity metric. Non-metric multidimensional scaling (NMDS) analysis was also used in our study to demonstrate horizontal distribution patterns of the bacterial communities in PRIMER V6.0 software (Clarke and Gorley, 2006). The bacterial community data were first square root transformed in the above software, and then, a lower triangular resemblance matrix was created based on the Bray–Curtis similarity.

The significant differences were evaluated by *t*-test in IBM SPSS Statistics 25. The scatter diagrams were conducted in Origin v8.5 and fitted by linear and exponential functions. Pearson correlations between ARGs, MGEs, and bacterial communities (at the phylum level) were computed using the “ggcorrplot” package and visualized by the “gglpot2” and “ggthemes” packages in the R v3.6.2 software.

## Results

### DNA Yield by Successive Extractions

**FIGURE 3:**
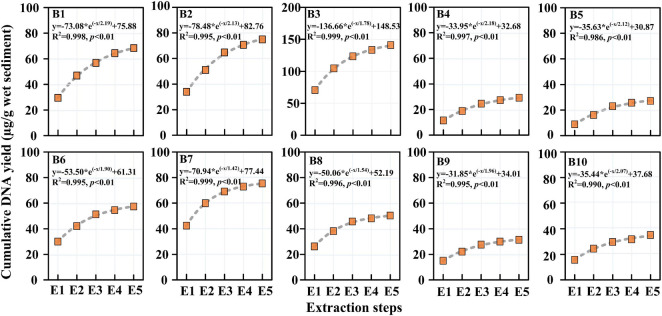
Accumulated DNA yields by successive DNA extraction from the 10 sediment samples. Note that the unit of the accumulated DNA yield is μg DNA/g wet sediment.

The DNA yields of sediment samples through successive DNA extractions are presented in Figure 3. The DNA yields of a single extraction ranged from 8.87 to 70.54 μg/g wet sediment, while the accumulated DNA yields ranged from 29.11 to 140.91 μg/g wet sediment. It can be calculated that the accumulated DNA yields increased 1.8–3.1 times compared with that of single DNA extraction. In the Haihe River, the DNA yield first increased and reached a maximum close to the urban city and then decreased until the Haihe estuary. The DNA yield in the Bohai Sea also showed the same trend as that in the Haihe River. The DNA yield by successive extraction reached a maximum at Sta. B3 and B7 in river and marine sediment samples, respectively. From the fitted curves, the DNA yields in all samples tended to plateau after five DNA extractions (Figure 3). There was a significant exponential relationship (*p* < 0.01) in all samples between accumulated DNA yields and the extraction cycles, indicating that the DNA yields were nearly close to the maximum after five succession extractions (Figure 3).

### Sequencing Analysis and Diversity Estimates

**FIGURE 4:**
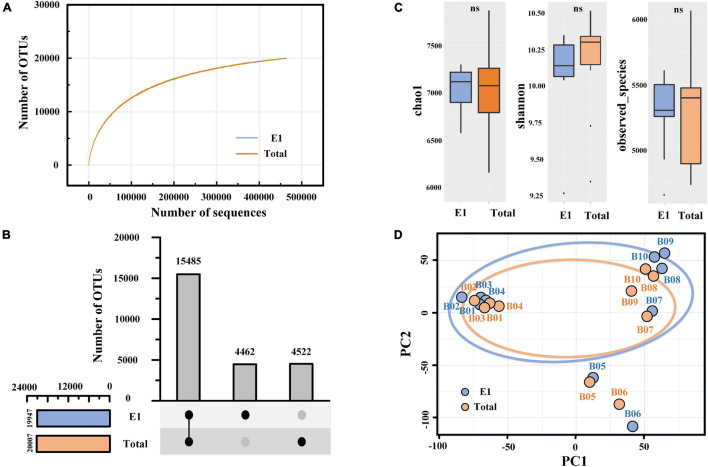
Alpha- and betadiversity of prokaryotic organisms revealed by 16S rRNA gene sequencing. **(A)** Rarefaction curves of similarity-based OTUs at 97% sequence similarity level of the two groups. **(B)** UpSet plot showing the shared and separated OTUs between the two groups. **(C)** Box plot showing the alpha diversity of the sequencing. One-way ANOVA was used to compare the differences between the two groups. **(D)** Principal component analysis (PCA) of bacterial communities based on Bray–Curtis distance. E1, single DNA extractions; total, combined samples of successive DNA extractions.

In the present study, the valid tags varied from 46,430 to 62,609 per sample after quality control and 46,430 per sample after resampling. Finally, the sequencing of 16S rRNA genes yielded 928,600 effective tags and 24,469 OTUs at 97% similarity. The total number of OTUs in all samples was typically higher than the diversity estimates, such as Chao1 (7,031 ± 454) and observed species (5,267 ± 347), revealing the huge difference between samples. As shown in Figure 4A, the rarefaction curves of the two groups (E1 and total) tended to plateau. Significantly, the OTU numbers in the total extraction (20,007 OTUs) were slightly higher than those in E1 (19,947 OTUs), and no significant difference was observed between the two groups, revealing that the extraction cycles had a negligible effect on the total species. The UpSet plot showed that the E1 and total samples shared 15,485 OTUs, while they had 4,462 and 4,522 separate OTUs, respectively (Figure 4B). These separated OTUs in the two groups mainly belonged to rare species, indicating that extraction cycles mainly affect rare species but have limited influence on abundant species. Similar to the OTU numbers, the alpha diversity index (Chao1, Shannon, and observed species) did not present a significant difference between the two groups (Figure 4C). Principal component analysis (PCA) showed that PCR bias and community structure differences existed between E1 and the total samples, especially in the marine sediment (Sta. B7–B10) (Figure 4D). In addition, we observed that the 10 sediment samples were divided into three groups in the PCA figure, including river sediments (Sta. B1–B4), estuary sediments (Sta. B5–B6), and marine sediments (Sta. B7–B10), implying that the bacterial communities varied in different sediment types.

### Species Composition and Diversity Changes

**FIGURE 5:**
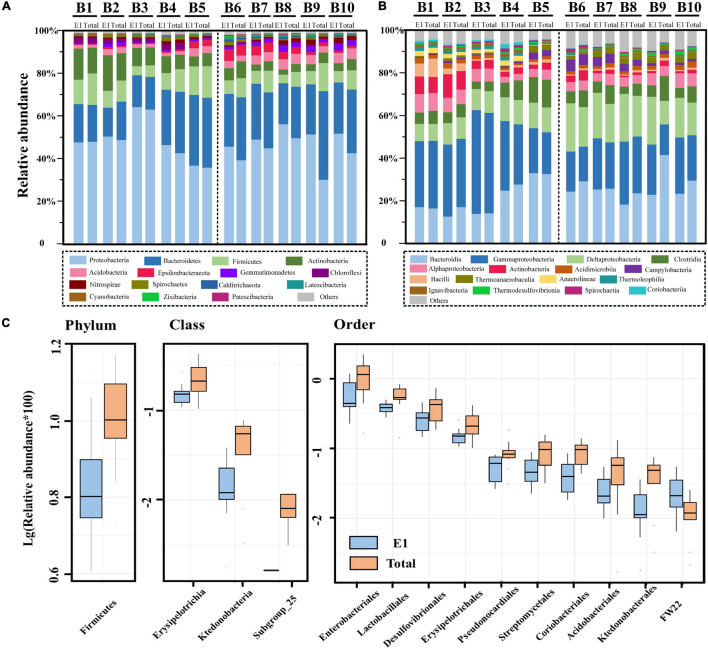
The relative abundance of bacterial communities by single DNA extractions (E1) and succession DNA extractions (total) in the river and marine sediment. **(A)** The relative abundance of bacterial communities at the phylum level. **(B)** The relative abundance of bacterial communities at the class level. **(C)** Box plot showing the significant difference species between two groups at the phylum, class, and order levels (top 10). Significant differences were compared by *t*-test, and only significantly different groups (*p* < 0.05) are shown in the figure.

The relative abundance of bacterial communities at the phylum level and class level are presented in Figures 5A,B, respectively. At the phylum level, Proteobacteria was the most diverse bacteria across all samples and all extraction strategies. However, there was no significant difference in Proteobacteria between the river and marine sediment, as well as the single and successive DNA extractions. The Proteobacteria in the present study were mainly composed of Alpha-, Gamma-, and Deltaproteobacteria, among which gammaproteobacteria dominated in both river and marine sediment. At the class level, the relative abundances of Alpha-, Gamma-, and Deltaproteobacteria were not significantly different between single and successive DNA extractions. However, at the order level, the Enterobacteriales within the class gamma-Proteobacteria and Desulfovibrionales within the class Deltaproteobacteria were all significantly different between single and successive DNA extractions (*p* < 0.05) (Figure 5C). We found that the relative abundance of Gammaproteobacteria was significantly different in river and marine sediments by successive DNA extractions (*p* = 0.046), while it was not significant in river and marine sediments by single DNA extraction (*p* = 0.097). Deltaproteobacteria showed a significant difference between river and marine sediments by both single DNA extraction (*p* < 0.01) and successive DNA extractions (*p* < 0.01). For Alphaproteobacteria, it was not significant between river and marine sediment by either single DNA extraction or successive DNA extractions.

Bacteroidetes was the second dominant group at the phylum level in all sediment samples. However, the *t*-test results showed that there was no significant difference between single and successive DNA extractions, as well as the river and marine sediments. Bacteroidetes was mainly composed of Bacteroidia and Ignavibacteria at the class level, among which Bacteroidia dominated. Firmicutes was the third dominant group at the phylum level across all samples. Firmicutes was mainly composed of Clostridia, Bacilli, and Erysipelotrichia. Among these, Erysipelotrichia was observed to have a significant difference between single and successive DNA extractions (*p* < 0.05), although it only accounted for a minor fraction (Figure 5C). In addition, the Lactobacillales within the class Bacilli also presented a significant difference between single and successive DNA extractions (*p* < 0.05) (Figure 5C). Actinobacteria and Acidobacteria were also commonly detected in the study region. The two groups presented different trends in the sediment: Actinobacteria was more dominant in the river sediment (*p* < 0.05 for both E1 and total), while Acidobacteria was more dominant in the marine sediment (*p* < 0.01 for E1 and *p* < 0.05 for total). However, we did not observe a significant difference in either Actinobacteria or Acidobacteria between the E1 and total samples. At the class level, Subgroup_25 within the class Acidobacteria was not detected in E1 samples but was sporadically detected in total samples. At the order level, Pseudonocardiales, Streptomycetales, and Coriobacteriales within the phylum Actinobacteria and Acidobacteriales within the phylum Acidobacteria all presented significant differences between single and successive DNA extractions (Figure 5C). Apart from the above five groups at the phylum level, Epsilonbacteraeota, Gemmatimonadetes, Chloroflexi, Nitrospirae, Spirochaetes, Calditrichaeota, Latescibacteria, Cyanobacteria, Zixibacteria, and Patescibacteria were also commonly detected in the study region. The *t*-test results showed that there were no significant differences in these groups at the phylum level between E1 and total samples. At the order level, Ktedonobacterales and FW22 within the phylum Chloroflexi showed significant differences between E1 and the total samples (*p* < 0.05) (Figure 5C).

### Abundance of Antibiotic Resistance Genes and *intl*1 in the Sediment

The selected nine ARGs were detected in all sediment samples, among which *sul*1 and qacEΔ1-01 had the highest absolute abundances. The total ARGs detected at high absolute abundances in each sample ranged from 2.17 × 10^6^ to 2.33 × 10^9^ copies/g by single DNA extraction and 8.74 × 10^6^–7.74 × 10^9^ copies/g by successive DNA extractions. The absolute abundances of total ARGs by successive DNA extractions were 1.3–4.0 times higher than those by single DNA extractions, revealing that ARGs were greatly underestimated by single DNA extractions. In addition, we found that the absolute abundances of *tet*M, *tet*W, bla_TEM_, and *erm*B by successive DNA extraction were typically higher than those by single DNA extraction, ranging from 1.0 to 11.1 times, 1.2 to 13.6 times, 3.4 to 16.0 times, and 2.6 to 26.7 times, respectively. The discrepancies between E1 and total samples of other ARG subtypes were all less than 5.0 times. The class 1 integron-integrase gene (*intl*1) was also measured in the present study, ranging from 1.33 × 10^6^ to 6.14 × 10^7^ copies/g by single DNA extraction and 3.08 × 10^6^–2.22 × 10^8^ copies/g by successive DNA extraction. The absolute abundances of *intl*1 by successive DNA extractions were 2.0–4.5 times higher than those by single DNA extractions.

**FIGURE 6:**
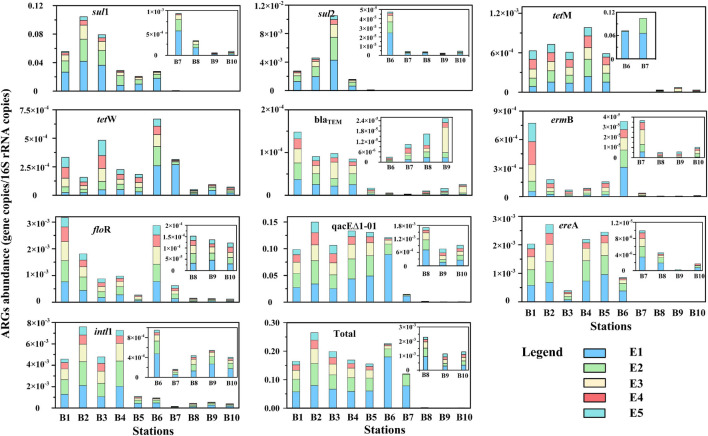
The relative abundance of antibiotic resistance genes (ARGs) and *intl*1 in different extract cycles from sediment samples. The absolute abundance of ARGs was standardized by 16S rRNA gene copies.

The relative abundances of ARGs and *intl*1 in the sediment samples are shown in Figure 6. The relative abundance of total ARGs ranged from 7.8 × 10^–4^ to 3.2 × 10^–1^ copies/16S rRNA gene copies and 1.1 × 10^–3^–2.6 × 10^–1^ copies/16S rRNA gene copies by single and successive DNA extractions, respectively. The relative abundances of total ARGs by successive DNA extractions were 1.3–4.0 times higher than those by single DNA extractions. The levels of the total ARGs in this study were comparable and even exceeded those reported in the same region by previous studies. Regionally, the sampling stations close to urban cities and estuaries had the highest total ARG abundance but dramatically decreased outward of Bohai Bay. In addition, we found that different subtypes of ARGs had different distribution trends. Sulfonamide resistance genes (*sul*1, *sul*2) were higher upstream of the Haihe River, especially at stations close to the main urban city of Tianjin (Sta. B2 and B3). Tetracycline resistance genes (*tet*M, *tet*W) were higher offshore of Bohai Bay (Sta. B5 and B6) than in other stations. Beta_lactamase resistance genes (bla_TEM_) were higher upstream of the Haihe River than in the estuary and Bohai Bay. Macrolide lincosamide–streptogramin B (*erm*B) and multidrug resistance gene (*flo*R) presented the same pattern that was significantly higher in Sta. B1 and B6 than other stations. The multidrug resistance gene qacEΔ1-01 was higher upstream and in the estuary of the Haihe River and dramatically decreased from the offshore to the open region of Bohai Bay.

### Linear Fitting of *intl*1 and Antibiotic Resistance Genes

**FIGURE 7:**
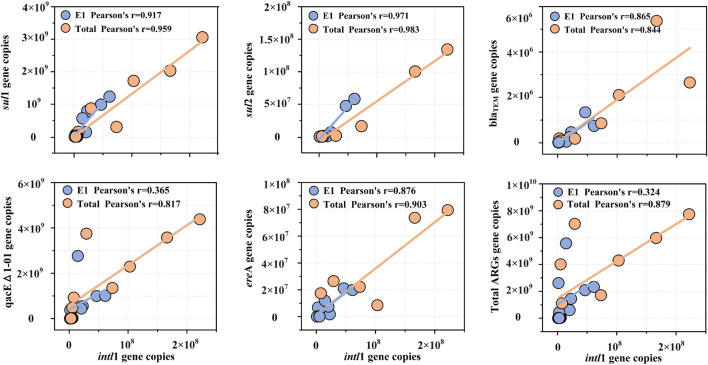
Correlation of five ARG subtypes (*sul*1, *sul*2, bla_TEM_, qacEΔ1-01, and *ere*A) and total ARGs with the class 1 integron-integrase gene (*intl*1). The absolute abundance of total ARGs was the sum of the absolute abundance of nine ARG subtypes measured in the present study. Note that only correlation coefficients (*R*) greater than 0.8 were included in the correlation analysis.

The linear fittings of *intl*1 and ARGs are shown in Figure 7. The absolute abundance of *intl*1 showed a high correlation coefficient with the absolute abundance of *sul*1, *sul*2, bla_TEM_, qacEΔ1-01, and *ere*A (*R* < 0.8) but presented a low fitting degree with *tet*M, *tet*W, *erm*B, and *flo*R (*R* < 0.8). The absolute abundance of *intl*1 showed the highest correlation coefficient with *sul*1 and *sul*2, implying that *intl*1 might be the main carrier of sulfonamide resistance genes. Although the correlation coefficient between *intl*1 and sulfonamide resistance genes (*r* = 0.917 for *sul*1, *r* = 0.971 for *sul*2) was high by single DNA extractions, the fitting correlation coefficient still further increased after five successive extractions (*r* = 0.959 for *sul*1, *r* = 0.983 for *sul*2). The fitting coefficient between bla_TEM_ and *intl*1 decreased slightly and was 0.865 for E1 samples and 0.844 for total samples. For qacEΔ1-01, the fitting coefficient with *intl*1 increased significantly for the two extraction strategies and was 0.365 for E1 samples and 0.817 for total samples. Macrolide lincosamide–streptogramin B (*ere*A) also showed a high fitting coefficient with *intl*1 for both extraction strategies and was 0.876 for E1 samples and 0.903 for total samples. The total abundance of ARGs was also fitted with the abundance of *intl*1, and the fitting coefficient increased significantly from 0.324 (single DNA extractions) to 0.879 (successive DNA extractions).

### Correlation Analysis of *intl*1, Antibiotic Resistance Genes, and Bacterial Communities

**FIGURE 8:**
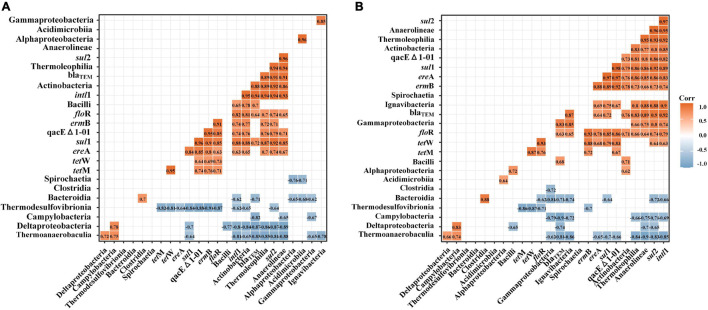
Pearson correlation analysis of the abundance of *intl*1, ARGs, and bacterial communities (top 14 at the class level) by single DNA extractions **(A)** and successive DNA extractions **(B)**. The abundances of ARGs, MGEs, and bacterial communities were log_10_ transformed before calculating Pearson’s correlation. Only strong (| *R*| > 0.6) and significant (*p* < 0.05) correlations are shown in the figure.

The correlations between *intl*1, ARGs, and bacterial communities differed widely between single and successive DNA extractions (Figure 8). In E1 samples, *sul*1 showed a significant positive correlation with Actinobacteria, Thermoleophilia, and Anerolineae and showed a significant negative correlation with Thermoanaerobaculia, Deltaproteobacteria, and Thermodesulfovibrionia. Compared with E1 samples, *sul*1 also showed significant positive correlations with Ignavibacteria in total samples. In addition, *sul*2 presented significant positive correlations with Actinobacteria and Thermoleophilia and showed significant negative correlations with Thermoanaerobaculia, Deltaproteobacteria, and Thermodesulfovibrionia in E1 samples. However, *sul*2 also presented significant positive correlations with Gammaproteobacteria, Ignavibacteria, and Anaerolineae and showed significant negative correlations with Campylobacteria. For tetM and tetW, the two subtypes showed the same correlation with bacteria and only showed significant negative correlations with Thermodesulfovibrionia. The beta_lactamase resistance genes (bla_TEM_) were positively correlated with Bacilli and negatively correlated with Bacteroidia, Campylobacteria, Deltaproteobacteria, and Thermoanaerobaculia in E1 samples. The correlation between bla_TEM_ and bacterial communities in total samples was almost the same as that in E1 samples but was also positively correlated with Gammaproteobacteria. qacEΔ1-01 did not show a positive correlation with bacterial communities in E1 samples but showed a significant positive correlation with Ignavibacteria. The two groups of samples all showed the same negative correlation with Thermodesulfovibrionia. The *flo*R gene only showed a significant negative correlation with Thermodesulfovibrionia in E1 samples but was also negatively correlated with Bacteroidia in total samples. For *erm*B, the two groups of samples showed the same correlation with bacterial communities and were only negatively correlated with Thermodesulfovibrionia. *ere*A showed a different negative correlation with bacterial communities in the two groups. It was negatively correlated with Thermodesulfovibrionia in E1 samples but showed a negative correlation with Thermoanaerobaculia. The universal class I integron-integrase gene (*int*I) showed grossly different correlations with bacterial communities between E1 and total samples. It only showed a significant positive correlation with Bacilli in E1 samples, whereas it also presented significant positive correlations with Anaerolineae, Thermoleophilia, Actinobacteria, and Ignavibacteria.

## Discussion

### Changes of DNA Yield by Successive Extraction

DNA extraction is a crucial step because it determines the accuracy of downstream molecular analysis, especially qPCR (Dimitrov et al., 2017). Previous studies have reported that DNA yield in multiple environmental mediums, such as soils and swine manure, was affected by successive DNA extractions (Feinstein et al., 2009; Dimitrov et al., 2017; Luo et al., 2021). To date, no study has been conducted to assess the DNA yield in sediment, although it is thought to be a hotspot for various pathogenic bacteria containing plentiful ARGs (Zhu et al., 2017; Zhao et al., 2020). Our results showed that the DNA yield was indeed affected by successive DNA extractions (Figure 3), which is in keeping with the assessment conducted in soil and swine manure (Feinstein et al., 2009; Luo et al., 2021). The fold changes (1.8–3.1 times) between single DNA extractions and successive DNA extractions in our study were roughly equivalent to a previous study conducted in soil samples, indicating that a significant portion of extractable DNA had been ignored in environmental samples by a commercial DNA extraction kit (Dimitrov et al., 2017). Two different causes may contribute to the elevated DNA yields in our study. On one hand, the repeated extraction of sediment samples in our study increased the beating time, which could help some insoluble cells lyse into the solution. Bürgmann et al. (2001) also reported that the beating time has a significant effect on the yield and quality of DNA extracted from soil samples. On the other hand, released DNA can be strongly adsorbed by particles in sediment samples, which can also influence the DNA yield (Lombard et al., 2011). Repeated elution by buffer can help DNA desorption from sediment components and finally increase the DNA yield (Lombard et al., 2011; Dimitrov et al., 2017).

The DNA yields in our study were highest at the first DNA extraction in all sediment samples and decreased with increasing extraction cycles. This phenomenon was in accordance with previous reports conducted in soil samples; however, it was different from a study conducted in swine manure (Feinstein et al., 2009; Jones et al., 2011; Luo et al., 2021). In addition, our results presented significant exponential relationships between accumulated DNA yields and extraction cycles (Figure 3), which is consistent with previous studies conducted in soil samples (Feinstein et al., 2009). Feinstein et al. (2009) suggested that bias can be adequately reduced in many situations by pooling three successive extractions in soil samples. However, the additional extraction cycles should be determined by different environmental media. For example, a different report observed a significant linear relationship between cumulative DNA yield and extraction cycles in swine manure, and the DNA yield still increased after six extraction cycles (Luo et al., 2021). The author explained that it could be the varied characteristics of samples such as the particle distribution and organic matter (Bürgmann et al., 2001). Our results showed that three and four successive extractions had approximately 83–91 and 91–97% of all extracted DNA recoveries, respectively. Considering the timeliness and economy, we suggested that three or four successive extractions should be added when extracting DNA from sediment samples to obtain higher DNA recovery.

### Underestimation of Antibiotic Resistance Genes and *intl*1 in Sediments

Whether in E1 or total samples, the total absolute abundance of ARGs (nine subtypes) in the Haihe Estuary (Sta. A5 and A6, Figure 6) in our study were typically higher than those in a previous study conducted in the same region (96 subtypes), revealing that the DNA extraction method could have a great influence on the abundance of ARGs (Zhao et al., 2020). The qacEΔ1-01 gene was the most abundant gene among the nine ARGs in the study region, which is consistent with the study in the Haihe Estuary, illustrating that the DNA extraction method has little influence on the dominant gene (Zhao et al., 2020). A recent continental-scale investigation of ARGs detected that multidrug resistance genes were highly diverse and abundant in coastal China (Zhu et al., 2017). Thus, it is a challenge to control infectious diseases and to avoid the widespread transmission of multidrug resistance under the prevalence of multidrug resistance genes in aquatic ecosystems (Oppegaard et al., 2001; Zhang et al., 2006). Similarly, sulfonamide resistance genes (*sul*1, *sul*2) also presented high absolute abundance in our study (Figure 6). The total absolute abundance of *sul*1 and *sul*2 in the sediment samples of the Haihe River (Sta. A1–A4) in this study were also higher than those in an earlier study in the same region after excluding several outliers, illustrating that sulfonamide resistance gene pollution has further intensified in recent years (Luo et al., 2010). In addition, we observed that the abundance of *sul*1 was higher than that of *sul*2 in our study, which was the opposite of a previous study (Luo et al., 2010). We observed that tetracycline resistance genes (*tet*M, *tet*W) were typically higher in the coastal region of Bohai Bay (Sta. A6 and A7) than other regions. We hypothesized that the high abundance of tetracycline resistance genes in coastal regions of Bohai Bay could be correlated with offshore aquaculture. A study found that *tet*M and *tet*S genes were present in fish intestinal and seawater bacteria at aquaculture sites through examination of isolated bacteria, and the author hypothesized that aquaculture could be an important reservoir of tetracycline resistance genes in the marine environment (Kim et al., 2004).

The absolute abundance of ARGs in the total samples was 1.3–4.0 times higher than that in the E1 samples, which was roughly equivalent to the fold changes of DNA yield (Figure 3). However, the enhanced abundance after additional extractions of a specific ARG subtypes showed huge differences between ARGs. According to the fitting curves, *tet*M, *tet*W, bla_TEM_, and *erm*B continued to have linear growth trends in most samples, revealing that their absolute abundances still did not reach a maximum after five successive extractions (Supplementary Figures 2, 4, 5). Unlike the above subtypes, *sul*1, *sul*2, qacEΔ1-01, *flo*R, *ere*A, and *intl*1 were well fitted by logarithmic equations in most samples, revealing that five successive extractions might be enough to quantify their abundance (Supplementary Figures 1, 3, 5). This indirectly showed that different ARG subtypes have different hosts, and these hosts may potentially be extracted in different periods of successive extractions. Luo et al. (2021) also reported that the fitting curves varied among different ARG subtypes in swine manure. However, our results showed that tetracycline resistance genes (*tet*M, *tet*W) continued to intensify after successive DNA extractions in sediment, while they were almost leveled off through six successive DNA extractions in swine manure (Luo et al., 2021). We speculated that the potential hosts of tetracycline resistance genes could be different between sediment samples and swine manure. For example, Gao et al. (2012) reported that most tetracycline resistance genes were isolated from *Bacillus* spp. in aquaculture environment. Another study reported that the tetM gene was detected in 34 Japanese and Korean isolates, including *Vibrio* sp., *Lactococcus garvieae*, and *Photobacterium damsela* subsp. *Piscicida* (Kim et al., 2004). However, *tet*M and *tet*W were significantly correlated with Bacteroidetes and Proteobacteria in swine manure, respectively (Luo et al., 2021). Another study reported that feeding *Bacillus coagulans* R11 to laying hens exposed to lead obviously increased the abundances of aminoglycoside and chloramphenicol ARGs (Xing et al., 2021).

The universal class I integron-integrase gene (*int*I) abundance was well fitted by a logarithmic equation in all samples in the present study (Supplementary Figure 6), which was distinct from the study in swine manure (Luo et al., 2021). The discrepancy could also be the different hosts in sediment samples and swine manure. Integron integrase gene sequences are reported to be positively correlated with the spread of antimicrobial resistance by facilitating lateral ARG transfer and incorporation into bacterial chromosomes (Gillings et al., 2008). Multiple studies have shown that the class I integron-integrase gene is a proxy for anthropogenic pollution and is allochthonous in water ecosystems (Gillings, 2014; Gillings et al., 2015). In the present study, the correlation coefficients (*R*) between ARGs and *intl*1 were all improved after five successive extractions (Figure 7). Thus, the modified extraction method could help to better understand and forecast horizontal gene transfer in aquatic ecosystems.

### Underestimation of the Relationship Between Bacterial Communities and Antibiotic Resistance Genes

Proteobacteria, Bacteroidetes, and Firmicutes were the most dominant groups in all sediment samples in the present study, and their dominance resembled earlier studies from a variety of coastal sediments (Wang et al., 2015; Zhao et al., 2020). However, the bacterial community structure was different between river, estuary, and marine sediments in the present study (Figure 4D). The difference in bacterial communities between samples could probably be induced by salinity, which has been verified by a previous study (Herlemann et al., 2011; Zhao et al., 2020). Among all bacterial communities at the class level, only Firmicutes showed significant differences between single DNA extractions and successive DNA extractions (Figure 5C). Firmicutes have also been reported to have huge differences between single and successive DNA extractions in soil samples and swine manure. Dimitrov et al. (2017) suggested that organisms belonging to Firmicutes are more difficult to lyse, which could be the result of their life strategy and/or morphological characteristics. Firmicutes is also known as a host for many ARGs. For example, a study reported that the *flo*R gene was detected in 26 strains (21.8%) of isolated Gram-negative Bacilli from freshwater salmon farms in Chile (Fernández-Alarcón et al., 2010). Erysipelotrichia within the phylum Firmicutes was significantly different between single and successive DNA extractions. However, there is no direct proof to show that Erysipelotrichia contains ARGs, although some species within this class have been proven to be pathogenic bacteria (Griffen et al., 2012; Chen and Jiang, 2014). Lactobacillales within the phylum Firmicutes also exhibited significant differences between single and successive DNA extractions in our study. Lactobacillales, an order of the class Bacilli within the phylum Firmicutes, was once reported to be resistant to vancomycin (Gad et al., 2013). At the order level, the Enterobacteriales within the class Gammaproteobacteria also showed a significant difference between single and successive DNA extractions in our study. Members of the order Enterobacteriales, such as pathogenic *Salmonella enterica* and *Escherichia coli*, are known to contain diverse ARG resistance to multiple antibiotics (Su et al., 2012; Singh et al., 2017). Thus, the result from high-throughput sequencing also means that the ARGs were underestimated by single DNA extraction-based qPCR analysis. Actinobacteria has also been reported as a host of ARGs by a previous study (Fatahi-Bafghi, 2019). For example, a study showed that a large number of Streptomyces strains within Streptomycetales isolated from soil were resistant to multiple antibiotics (Nikaido, 2009). Our results also showed that Streptomycetales were significantly different between single and successive extractions (Figure 5C).

Previous studies have used co-occurrence networks to show the relationship between ARGs, MGEs, and bacterial communities and to forecast the potential hosts of ARGs (Zhu et al., 2017; Zhao et al., 2020). The first step of network construction is to calculate the correlation coefficient and corresponding *p*-value (Zhu et al., 2017; Zhao et al., 2020). Here, we used a more intuitive method to show the correlation difference between single and successive DNA extraction samples. The present study showed that there were more correlations between ARGs and ARGs, ARGs and MGEs, ARGs and bacterial communities in the successive DNA extraction samples than that in the single DNA extraction samples, which means that many potential hosts of ARGs were not identified by single DNA extractions (Figure 8). Presumably, the increased correlations ascribed to the lysis of insoluble pathogenic bacteria and DNA desorption from particles could alter the structure of bacterial communities and increase the abundance of ARGs. The greater correlations between ARGs in successive DNA extraction samples illustrated that these genes may be located in the same genetic elements or carried by specific bacterial species (Han et al., 2017). MGEs have been confirmed to play an important role in the persistence and proliferation of ARGs via horizontal gene transfer (Zhao et al., 2020). The greater correlations between ARGs and MGEs in successive DNA extraction samples suggested that the horizontal gene transfer of AGRs might be ignored by single DNA extraction, especially for tetracycline resistance genes and beta_lactamase resistance genes.

Bacterial communities were the main factor that directly affected the distribution of ARGs in the environment because ARGs consist of bacterial cells. Thus, the significant correlations between ARGs and bacterial communities suggested that the bacterial communities might be possible ARG hosts (Wang et al., 2020). We found that the correlations between ARGs and bacterial communities were more robust in the total samples than in the E1 sample, indicating that their interactive relationships greatly improved (Figure 8). Forsberg et al. (2014) suggested that the abundance and distribution characteristics of ARGs and bacterial communities were similar if they showed strong and positive correlations. This result may provide new insight into ARGs and their potential hosts in sediments. Significant negative correlations were also observed between ARGs and bacterial communities in the present study, which indicates that these classes probably impact the abundance and diversity of ARGs in sediments. In addition, we also found many potential hosts of ARGs that were previously not observed. For example, we found that *sul*1 also showed a significant correlation with Ignavibacteria in total samples. However, none of the previous studies have demonstrated that species within the class of Ignavibacteria are potential hosts of *sul*1 (Zhu et al., 2017; Zhao et al., 2020). However, correlation analysis is an indirect method to prove potential hosts of ARGs. The direct way to prove hosts of ARGs is to isolate bacteria by coating plates containing antibiotics. Further investigations on niche specialization and ecophysiological characterization of ARG-carrying pathogenic bacteria are greatly needed to better understand the interactions of ARGs, MGEs, and bacterial communities.

## Conclusion

Taken together, our study demonstrated that single DNA extraction of sediment samples by a commercial kit could result in gross underestimation of the abundance of ARGs and MGEs. Simultaneously, insufficient DNA extraction can also generate PCR bias, skew bacterial community structure, and underestimate some ARG-carrying pathogenic bacteria in sediments. It is expected that the elevated DNA extraction cycles could help better understand the risk and spread of ARGs and their correlation with bacterial communities in sediments. However, different subtypes of ARGs and MGEs did not respond equally to the additional extraction cycles, indicating that additional rounds of DNA extraction might still not be enough for the detection of some specific ARGs and MGEs in sediment samples. Further studies based on high-throughput quantitative PCR and metagenomic sequencing are greatly needed to better understand the extraction efficiency of more ARGs and MGEs in sediments and other environmental media.

## Data Availability Statement

The datasets presented in this study can be found in online repositories. The names of the repository/repositories and accession number(s) can be found below: https://www.ncbi.nlm.nih.gov/bioproject/PRJNA724916, PRJNA724916.

## Author Contributions

JS designed the experimental scheme and article framework and did the manuscript revision. CW wrote the manuscript. GZ, WX, SJ, LP, and DJ attended the cruises and did the sampling work. All authors contributed to the article and approved the submitted version.

## Conflict of Interest

The authors declare that the research was conducted in the absence of any commercial or financial relationships that could be construed as a potential conflict of interest.

## Publisher’s Note

All claims expressed in this article are solely those of the authors and do not necessarily represent those of their affiliated organizations, or those of the publisher, the editors and the reviewers. Any product that may be evaluated in this article, or claim that may be made by its manufacturer, is not guaranteed or endorsed by the publisher.

## Supplementary Material

Supplementary Figure 1 Cumulation abundance of sulfonamide resistance genes by successive DNA extraction from sediments. **(A)** Cumulation abundance of *sul*1. **(B)** Cumulation abundance of *sul*2.

Supplementary Figure 2 Cumulation abundance of tetracycline resistance genes by successive DNA extraction from sediments. **(A)** Cumulation abundance of *tet*M. **(B)** Cumulation abundance of *tet*W.

Supplementary Figure 3 Cumulation abundance of multidrug resistance genes by successive DNA extraction from sediments. **(A)** Cumulation abundance of qacEΔ1-01. **(B)** Cumulation abundance of *flo*R.

Supplementary Figure 4 Cumulation abundance of beta_lactamase resistance genes (bla_TEM_) by successive DNA extraction from sediments.

Supplementary Figure 5 Cumulation abundance of macrolide lincosamide-streptogramin B genes by successive DNA extraction from sediments. **(A)** Cumulation abundance of *ere*A. **(B)** Cumulation abundance of *erm*B.

Supplementary Figure 6 Cumulation abundance of the universal class I integron-integrase gene (*int*I) by successive DNA extraction from sediments.

Supplementary Table 1 The primer sequences for the targeted ARGs, MGEs and 16S rRNA gene and their classification by antibiotic target and resistance mechanism.
