# Supplementary figures and images for: New Estimation of Antibiotic Resistance Genes in Sediment Along the Haihe River and Bohai Bay in China: A Comparison Between Single and Successive DNA Extraction Methods
Source: Front Microbiol. 2021 Sep 20;12:705724. doi: 10.3389/fmicb.2021.705724 (PMC8488291; doi:10.3389/fmicb.2021.705724)

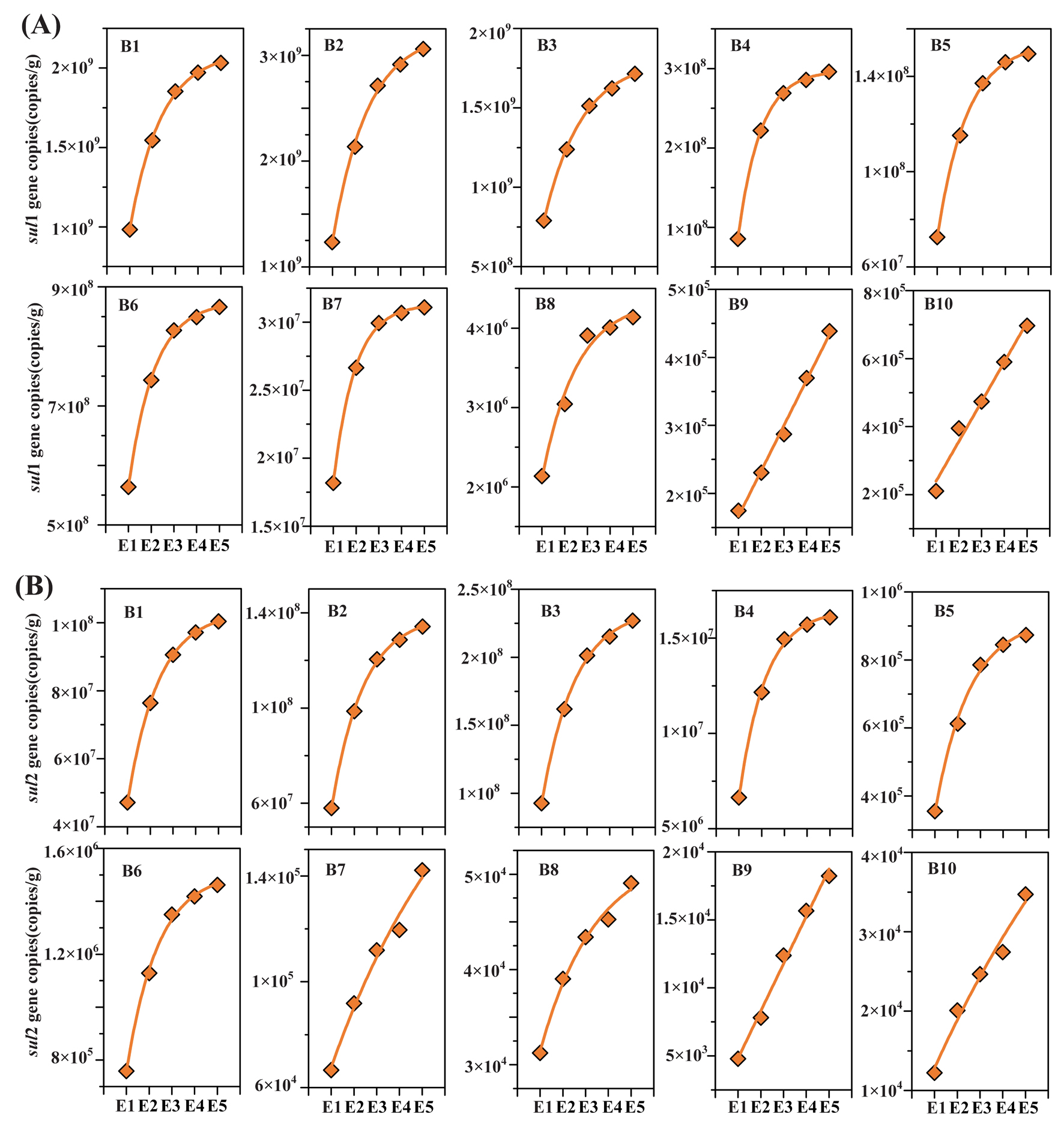

Supplement: Supplementary Figure 1 — Cumulation abundance of sulfonamide resistance genes by successive DNA extraction from sediments. (A) Cumulation abundance of sul1. (B) Cumulation abundance of sul2. [file Image_1.JPEG]

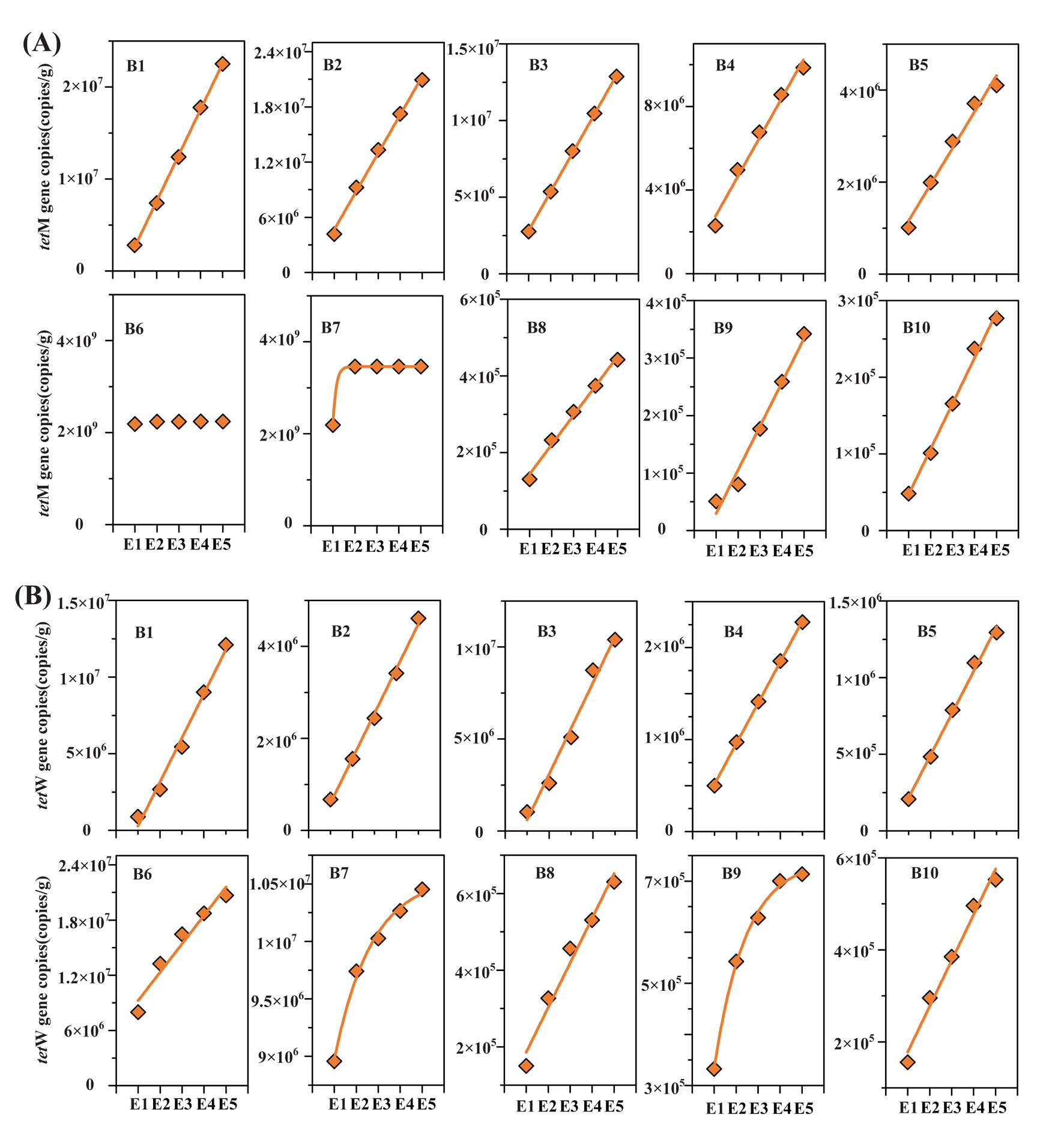

Supplement: Supplementary Figure 2 — Cumulation abundance of tetracycline resistance genes by successive DNA extraction from sediments. (A) Cumulation abundance of tetM. (B) Cumulation abundance of tetW. [file Image_2.JPEG]

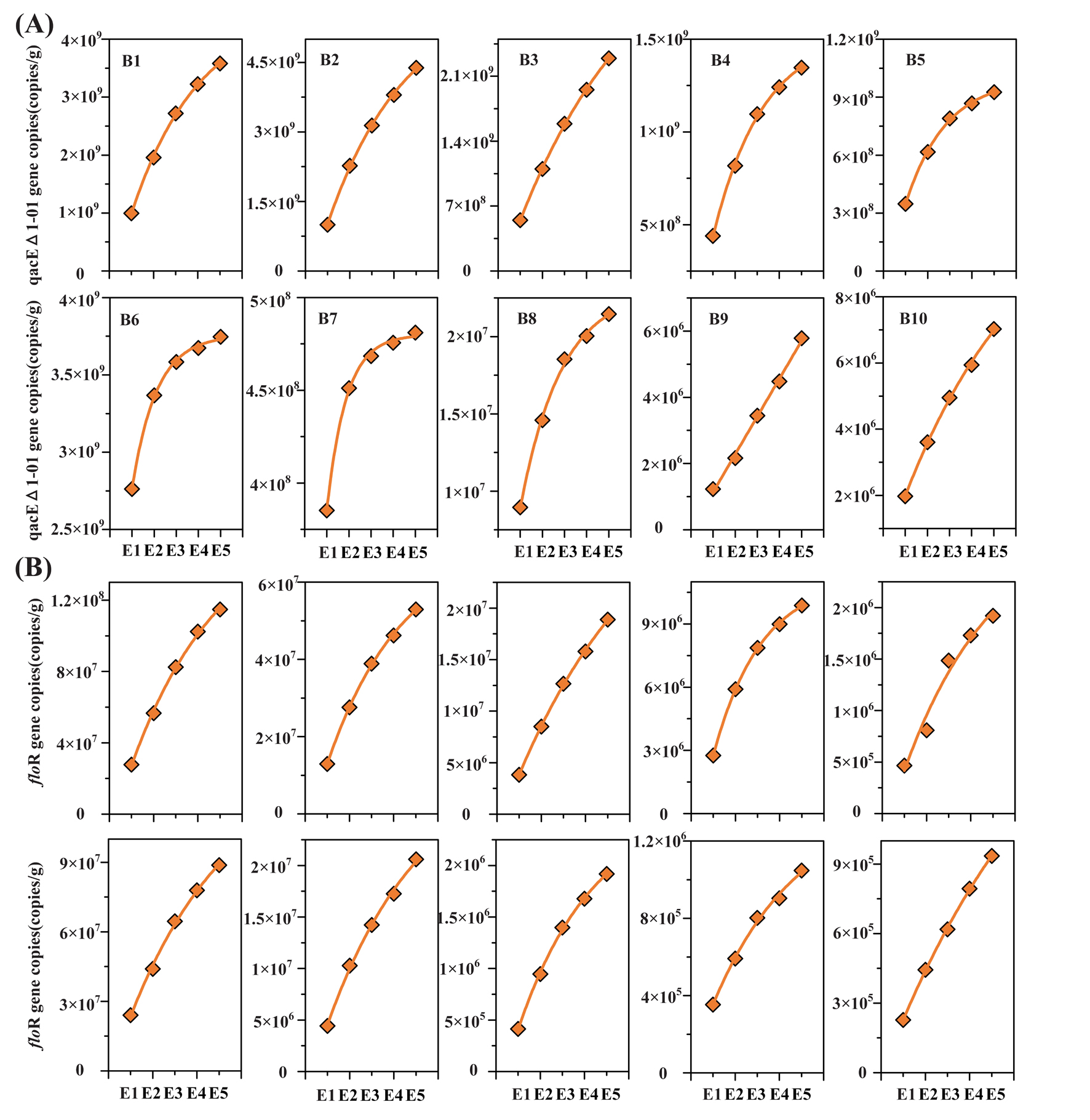

Supplement: Supplementary Figure 3 — Cumulation abundance of multidrug resistance genes by successive DNA extraction from sediments. (A) Cumulation abundance of qacEΔ1-01. (B) Cumulation abundance of floR. [file Image_3.JPEG]

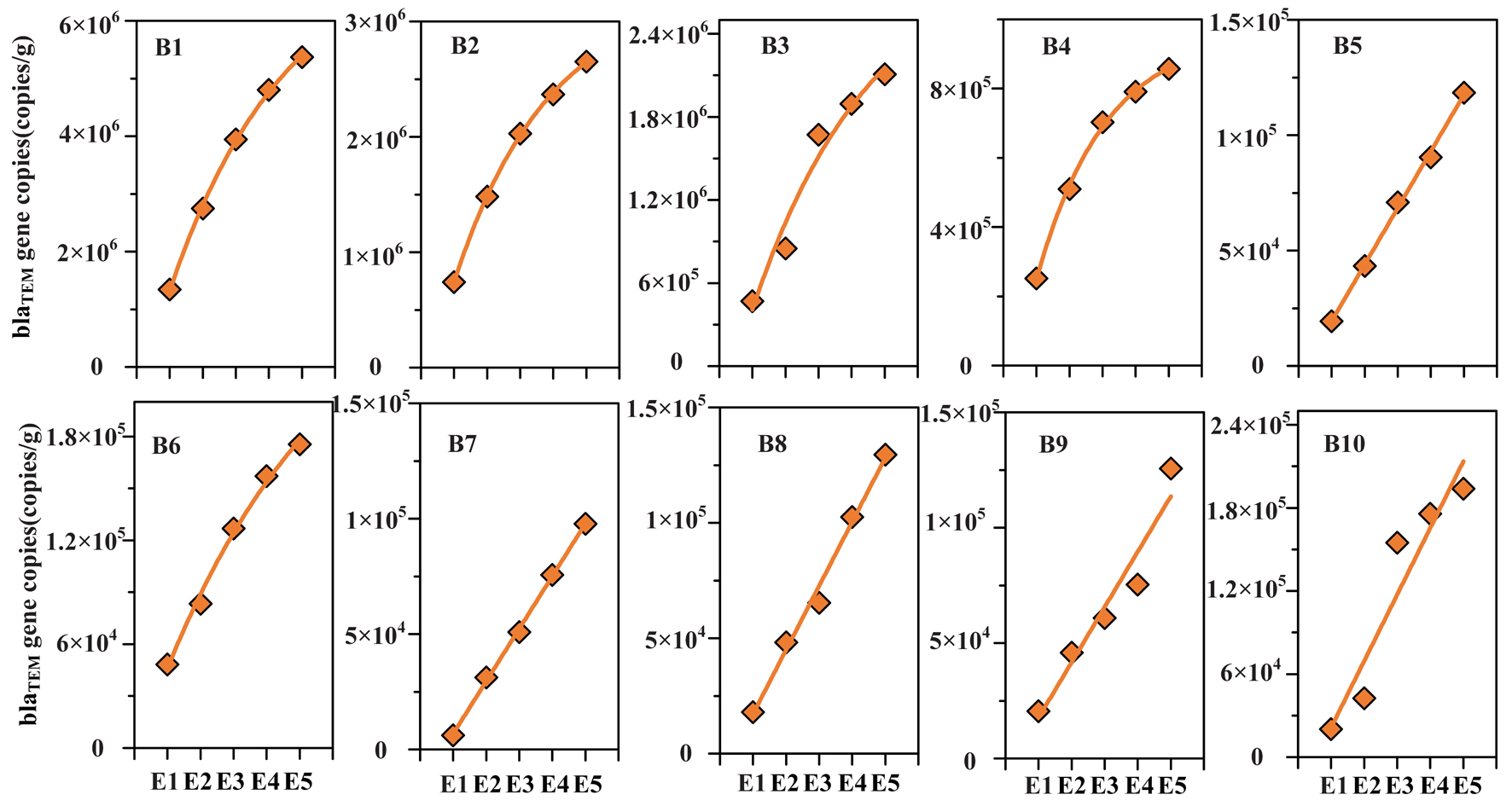

Supplement: Supplementary Figure 4 — Cumulation abundance of beta_lactamase resistance genes (blaTEM) by successive DNA extraction from sediments. [file Image_4.JPEG]

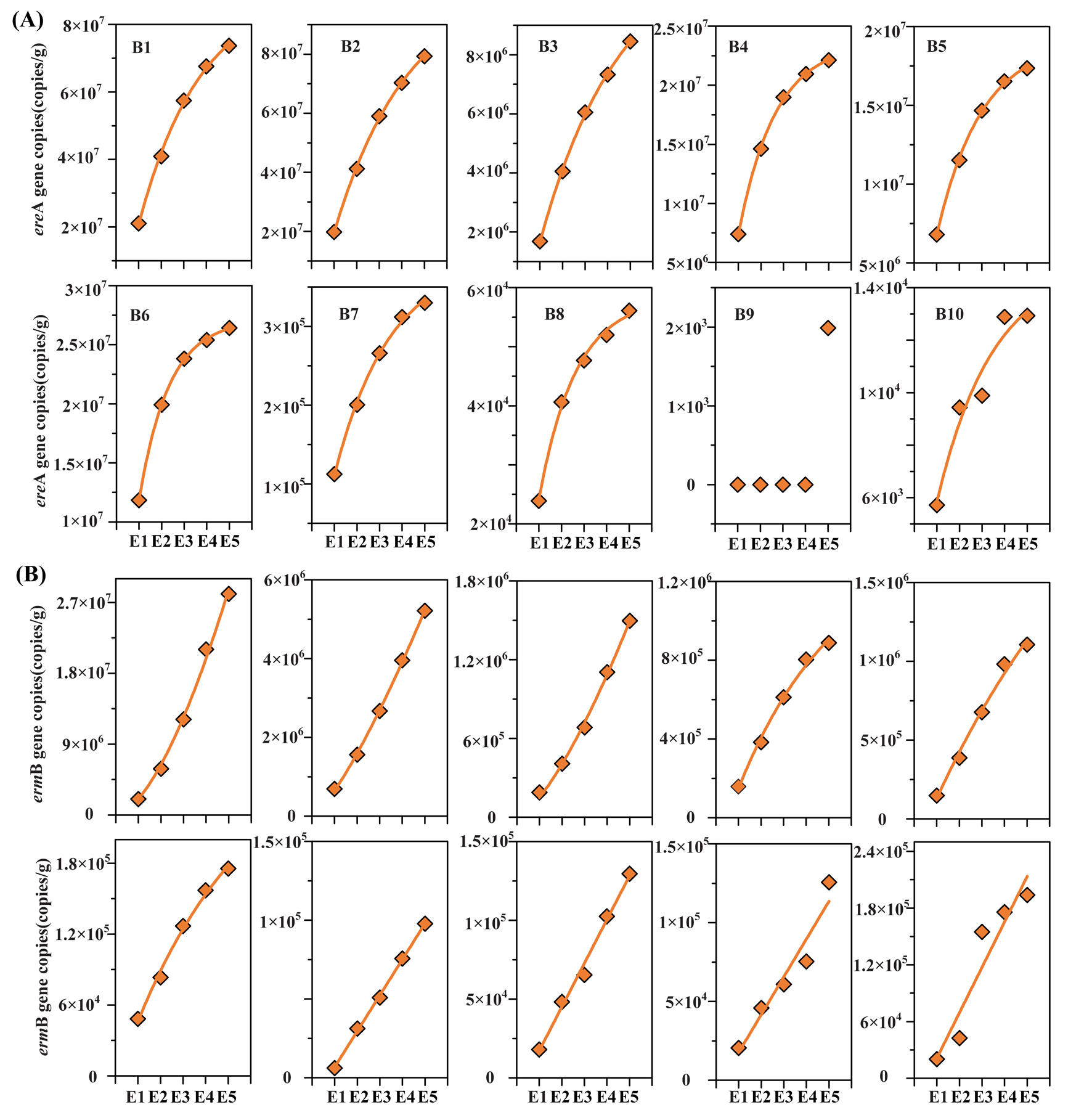

Supplement: Supplementary Figure 5 — Cumulation abundance of macrolide lincosamide-streptogramin B genes by successive DNA extraction from sediments. (A) Cumulation abundance of ereA. (B) Cumulation abundance of ermB. [file Image_5.JPEG]

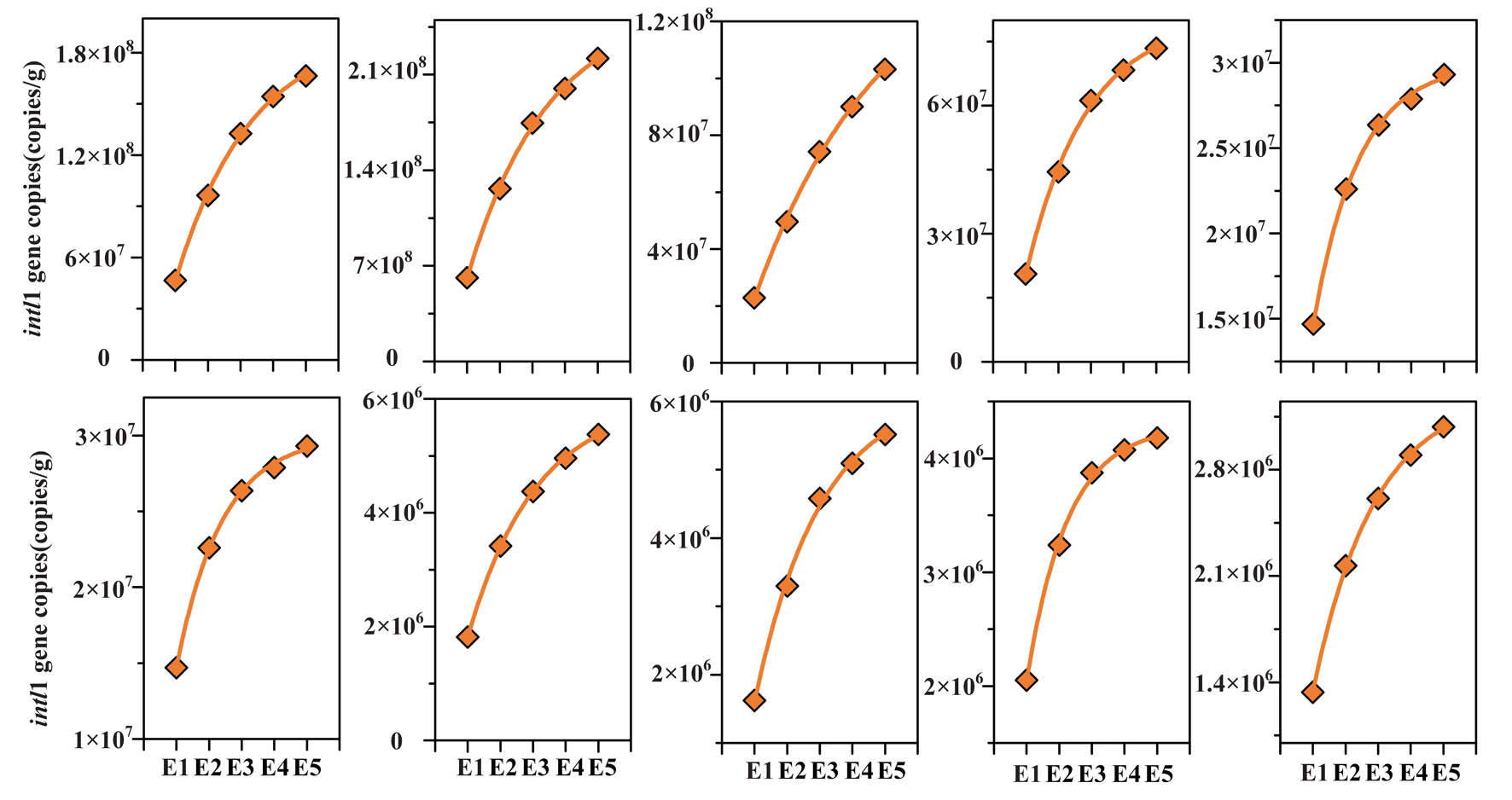

Supplement: Supplementary Figure 6 — Cumulation abundance of the universal class I integron-integrase gene (intI) by successive DNA extraction from sediments. [file Image_6.JPEG]
