# Supplementary material for: New Estimation of Antibiotic Resistance Genes in Sediment Along the Haihe River and Bohai Bay in China: A Comparison Between Single and Successive DNA Extraction Methods
Source: Front Microbiol. 2021 Sep 20;12:705724. doi: 10.3389/fmicb.2021.705724 (PMC8488291; doi:10.3389/fmicb.2021.705724)
Supplement: Supplementary Table 1 — The primer sequences for the targeted ARGs, MGEs and 16S rRNA gene and their classification by antibiotic target and resistance mechanism. [file Table_1.docx]

**Supplementary Table 1 The primer sequences for the targeted ARGs, MGEs and 16S rRNA gene and their classification by antibiotic target and resistance mechanism.**

| Gene Name | Forward Primer | Reverse Primer | Annealing temperature (℃) | Gene Classification | Resistance Mechanism |
| --- | --- | --- | --- | --- | --- |
| 16s rRNA | GGGTTGCGCTCGTTGC | ATGGYTGTCGTCAGCTCGTG | 62 | 16S rRNA | NA |
| *intI*1 | CGAACGAGTGGCGGAGGGTG | TACCCGAGAGCTTGGCACCCA | 60 | MGEs/Integrase | integrase |
| *sul*1 | CACCGGAAACATCGCTGCA | AAGTTCCGCCGCAAGGCT | 60 | Sulfonamide | cellular protection |
| *sul*2 | CTCCGATGGAGGCCGGTAT | GGGAATGCCATCTGCCTTGA | 60 | Sulfonamide | cellular protection |
| bla_TEM_ | AGCATCTTACGGATGGCATGA | TCCTCCGATCGTTGTCAGAAGT | 56 | β-lactam | antibiotic deactivation |
| *tet*M | CATCATAGACACGCCAGGACATAT | CGCCATCTTTTGCAGAAATCA | 55 | Tetracycline | cellular protection |
| *tet*W | ATGAACATTCCCACCGTTATCTTT | ATATCGGCGGAGAGCTTATCC | 54 | Tetracycline | cellular protection |
| *erm*B | TAAAGGGCATTTAACGACGAAACT | TTTATACCTCTGTTTGTTAGGGAATTGAA | 54 | MLSB | cellular protection |
| *ere*A | CCTGTGGTACGGAGAATTCATGT | ACCGCATTCGCTTTGCTT | 60 | MLSB | antibiotic deactivation |
| qacE∆1-01 | CCCCTTCCGCCGTTGT | CGACCAGACTGCATAAGCAACA | 60 | Multidrug | efflux pump |
| *flo*R | ATTGTCTTCACGGTGTCCGTTA | CCGCGATGTCGTCGAACT | 60 | Multidrug | efflux pump |
